# A Niobium Coordination Polymer as an Efficient Sorbent for Caffeine Detection in Surface Water

**DOI:** 10.1021/acsomega.5c04894

**Published:** 2025-08-21

**Authors:** Emily Pachêco Squizatto, Iare S. Ribeiro, Marcos V. S. Pereira, Fábio Junior M. Novaes, Márcio J. da Silva, Luciano G. Moura, Gilberto R. da Silva Junior, Renê C. da Silva, Jemmyson R. de Jesus

**Affiliations:** † Research Laboratory in bionanomaterials, LPbio, Department of Chemistry, Federal University of Viçosa, 36570-900 Viçosa, Minas Gerais, Brazil; ‡ Analytical Chemistry Laboratory − LAQUA, Federal University of Viçosa, 36570-900 Viçosa, Minas Gerais, Brazil; § Department of Chemistry, 28120 Federal University of Viçosa, 36570-900 Viçosa, Minas Gerais, Brazil; ∥ Department of Physics, Federal University of Viçosa, 36570-900 Viçosa, Minas Gerais, Brazil

## Abstract

Herein, it is reported the synthesis of a niobium-based metal–organic framework (MOF), [Nb­(Bez­(COO)_2_)]_*n*_, for the extraction of caffeine from surface waters. The material was synthesized and characterized by Fourier-transform infrared spectroscopy (FTIR), Raman spectroscopy, scanning electron microscopy (SEM), X-ray diffraction (XRD), and Brunauer–Emmett–Teller (BET) analysis, which confirmed the coordination between the ligand (1,4-benzenodicarboxylic, (Bez­(COO)_2_)) and niobium (Nb) with a morphology composed of hexagonal rods, high crystallinity, and a surface area of 94.7 m^2^ g^–1^. The extraction process was optimized using a response surface methodology, evaluating three factors: (i) mass of the MOF (100–500 mg), (ii) solution pH (5.0–9.0), and (iii) temperature (25–45 °C). The optimal conditions for caffeine extraction were determined as 10 mg of material, pH 9.0, and temperature of 25 °C. Adsorption studies showed that the Freundlich isotherm model provided the best fit (*R* ^2^ = 0.9498), suggesting adsorption on a heterogeneous surface. Kinetic studies showed that the intraparticle diffusion model better described the adsorption process (*R* ^2^ = 0.9554), highlighting physisorption by intraparticle diffusion as the predominant mechanism. Thermodynamic parameters revealed spontaneous and exothermic adsorption, with Δ*G* values between −5.052 and −4.668 kJ mol^–1^. The developed analytical method showed a linear range from 1.0 to 20 μg mL^–1^, with good linearity (*R* ^2^ = 0.9978), a limit of detection and quantification of 0.54 μg mL^–1^, and 1.78 μg mL^–1^, respectively. Accuracy was confirmed by recovery of 95.6 ± 1.1% at 4.5 μg mL^–1^. Moreover, the [Nb­(Bez­(COO)_2_)]_*n*_ material demonstrated high reusability, maintaining its extraction efficiency after five consecutive adsorption–desorption cycles. These results confirm the robustness, efficiency, and sustainability of [Nb­(Bez­(COO)_2_)]_*n*_ for environmental monitoring and remediation applications.

## Graphical abstract

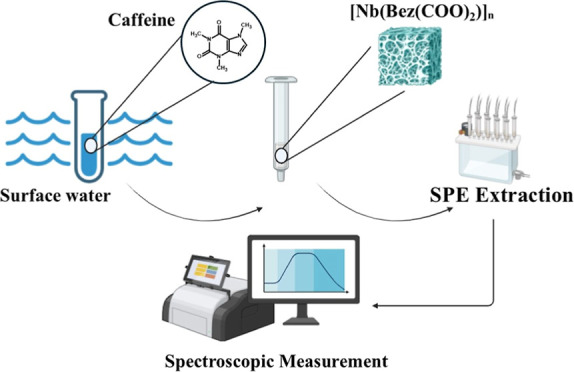

## Introduction

The disorderly growth of coastal urban centers has intensified pressure on aquatic ecosystems, especially in regions where basic sanitation is precarious or nonexistent.^1^ Inadequate discharge of domestic sewage and untreated wastewater represents one of the main sources of contamination of water bodies, compromising environmental quality and public health.^2^ In this context, the identification of anthropogenic contaminants has proven to be an effective strategy for tracking diffuse sources of pollution, with molecular markers being promising tools for this purpose.^1^

In recent years, numerous studies have reported the presence of several emerging contaminants in aquatic environments worldwide, including pharmaceutical compounds, personal care products, endocrine disrupting substance, illegal drugs, among others.^3^ Among the most frequently detected substances is caffeine (1,3,7-trimethylxanthine, Figure 1), a compound that has generated particular concern due to its persistence and biological activity. Caffeine, a central nervous system stimulant, is commonly found in beverages like coffee, tea, soft drinks, and energy drinks, making it one of the most widely ingested pharmaceutical substances, with an average intake of 177.7 mg per person per day.^3–5^

**Figure 1:**
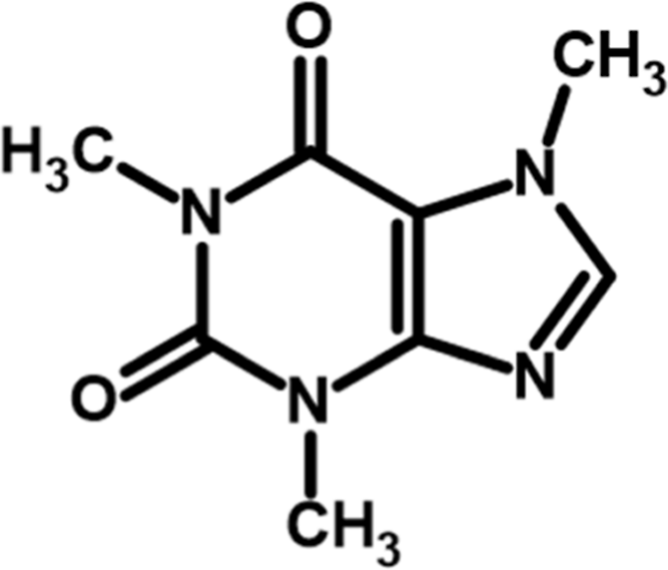
Chemical structure of caffeine (1,3,7-trimethylxanthine).

Although human metabolism considerably reduces caffeine levels in urine, direct disposal of caffeine-containing waste and washing of utensils still contributes to its release into sewage systems.^6^ The presence of caffeine in aquatic environments is of concern due to its physicochemical properties and potential for chronic toxicity, posing risks to aquatic organisms.^2^ Furthermore, the detection of caffeine in water bodies is often considered an indicator of domestic wastewater contamination.^1^ Despite its environmental importance, the identification of caffeine at typical environmental concentrations (usually in parts per million or less) remains analytically challenging, requiring a prior sample preparation step.^5^

Several water remediation technologies, including reverse osmosis and nanofiltration, have been proposed for the removal of emerging contaminants from aquatic environments.^7^ However, these methods are often limited by high operational costs. Alternative approaches, such as photodegradation and advanced oxidation processes (AOP), have also been explored for the degradation of pharmaceuticals and personal care products (PPCPs) in water bodies. Despite their potential, these techniques still face challenges related to efficiency, operational complexity, and energy demand. In this context, adsorption remains a widely adopted strategy due to its simplicity, high removal efficiency, low cost, operational feasibility under mild conditions, and wide range of available adsorbent materials.^7^ Several adsorbents have been extensively investigated for the removal of emergent contaminants from water, including activated carbon,^8^ biochar,^9^ zeolite,^10^ perovskites, and metal–organic frameworks (MOFs).^7^ Among them, MOFs offer distinct advantages such as high surface area, tunable pore structure, and high selectivity, making them a promising alternative for efficient extraction and preconcentration of contaminants in environmental matrices.^11–15^

In addition to their excellent physicochemical properties, MOFs exhibit excellent chemical and mechanical stability, allowing multiple reuse cycles without significant loss of adsorption capacity or structural degradation.^16^ This characteristic is in line with the principles of White Analytical Chemistry (WAC), a comprehensive and integrative framework that evaluates analytical methods based on three pillars, analytical performance (red), operational feasibility (blue), and environmental sustainability (green).^17,18^ WAC represents a conceptual advance over Green Analytical Chemistry, incorporating not only ecological considerations but also the efficiency and accessibility of analytical technologies.^18^

In line with the principles of WAC, this study proposes the development of a simple, efficient and ecofriendly method for the extraction and quantification of caffeine in surface waters, employing a niobium-based MOF as adsorbent material. The use of niobium is particularly innovative, since this element has been underutilized in MOF synthesis, despite its low toxicity, high thermal and chemical stability in aqueous environments and strong potential for the development of robust materials for environmental monitoring. To the best of our knowledge, this is one of the first studies to explore a niobium-terephthalate coordination polymer for caffeine removal, highlighting its application in water analysis as original and promising.

## Experimental Section

### Reagents

Ethylene glycol was purchased from Sigma-Aldrich (99%, Steinheim, Germany). Ammonium niobium oxalate was sourced from CBMM (MG, Brazil). 1,4-benzenedicarboxylic acid (Bez­(COOH_2_)_2_) was obtained from Sigma-Aldrich (98%, Steinheim, Germany). Sodium hydroxide (NaOH) was obtained from VETEC (97%, RJ, Brazil). Ultrapure water was produced using a Milli-Q system (Bedford, USA). Caffeine was purchased from Beantown Chemical (99%, Hudson, USA). For buffer solution preparation, monohydrated citric acid and disodium phosphate were obtained from Dinâmica (99%, SP, Brazil), monohydrated potassium citrate and anhydrous sodium carbonate from VETEC (99% RJ, Brazil), sodium bicarbonate from ISOFAR (99% RJ, Brazil), and monosodium phosphate monohydrate from MERCK (90%, RJ, Brazil). All reagents and materials were used as received, without further purification. Additionally, during extraction process, 5.00 mL Luer Slip cartridges (Descarpack) and glass wool (Perfyl Tech, Brazil) were employed for sealing, preventing material loss during sample extraction.

### Sample Collection

In October 2024, three surface water samples (totaling 750.0 mL) were obtained from the lagoon located on the campus of the Federal University of Viçosa (UFV), in Minas Gerais, Brazil at approximately 20°45′37″S, 42°52′04″W. Sampling included open water regions and areas suspected of being influenced by domestic wastewater discharge. Each sample was collected using 1 L amber glass bottles that had been previously decontaminated, ensuring collection at a depth of approximately 30 cm to avoid interference from the surface microlayer. After collection, water samples were immediately stored under refrigerated conditions (<4 °C) and protected from light to preserve their integrity. In cases where rapid extraction was not possible, samples remained refrigerated under the same conditions until additional analytical procedures were performed.

### Stock Solutions

To perform the extractions, working stock solutions of caffeine were initially prepared at a concentration of 200 μg mL^–1^ in deionized water. From these, diluted solutions at 100 μg mL^–1^ were subsequently prepared for use in the extraction procedures. The calibration curve was constructed using caffeine standards in the concentration range of 1.0 to 20.0 μg mL^–1^.

### Synthesis of [Nb­(Bez­(COO)_2_)]_*n*_

The MOF was synthesized via a solvothermal method as described by Squizzatto et al.,^19^ with minor modification in the stoichiometric ratios between the organic ligand 1,4-benzenedicarboxylic acid (Bez­(COOH_2_)_2_) and the Nb precursor. The synthesis was carried out in two distinct stages (Scheme 1). Initially, 1.50 g of Bez­(COOH_2_)_2_ were dissolved in 10.00 mL of deionized water at room temperature. After complete dissolution, the pH of the solution was adjusted to 12 using an NaOH solution (8 mol L^–1^) to promote complete deprotonation of the ligand carboxyl groups. Then, 0.50 g of ammonium niobium oxalate were gradually added to the solution under continuous stirring until complete homogenization. The resulting mixture was transferred to a Teflon-lined stainless-steel autoclave, followed by the addition of 4.00 mL of ethylene glycol, acting as a cosolvent and structure-directing agent. The autoclave was sealed and subjected to solvothermal conditions at 200 °C for 24 h. After the heating period, the system was allowed to cool naturally to room temperature. The obtained precipitate was collected and thoroughly washed three times with Milli-Q water and once with ethanol to remove any unreacted precursors and byproducts. Each washing step involved centrifugation at 4,000 rpm for 10 min. Finally, the solid product was dried in a conventional oven at 50 °C for 8 h to obtain the final MOF, [Nb­(Bez­(COO)_2_)]_*n*_.

**Scheme 1:**
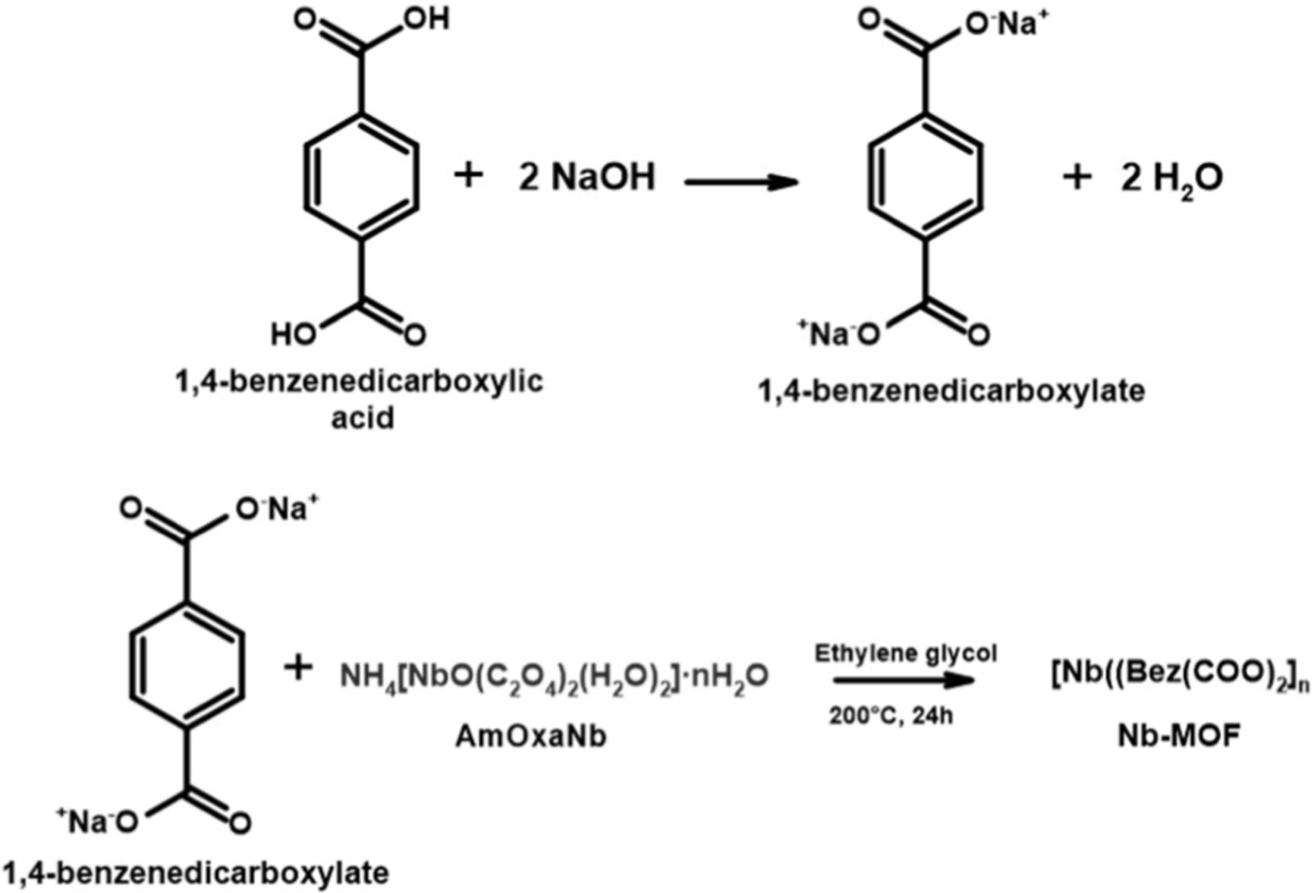
General Scheme Illustrating the Synthesis of [Nb­(Bez­(COO)_2_)]_*n*_

### Haracterization of [Nb­(Bez­(COO)_2_)]_*n*_

The compound [Nb­(Bez­(COO)_2_)]_*n*_ was characterized by several instrumental techniques. Nitrogen adsorption and desorption isotherms were obtained using a Nova 600 Series analyzer (Anton Paar, Graz, Austria), and the specific surface area was determined by the Brunauer–Emmett–Teller (BET) method. Raman spectroscopy was performed on a MicroRaman system (Renishaw, Wotton-under-Edge, United Kingdom), using a 633 nm laser with a power of 3 mW and an integration time of 30 s. The analyses were performed in quintuplicate to ensure data reproducibility. The thermal stability of the material was evaluated by thermogravimetric analysis (TGA). A thermobalance (PerkinElmer, USA) was used with a temperature range of 25 to 900 °C, under a nitrogen atmosphere and a heating rate of 10 °C/min. Morphological analyses were performed using scanning electron microscopy (SEM) on a JEOL JSM-6010LA microscope equipped with Everhart-Thornley and solid-state detectors, which allowed images to be obtained with contrasts associated with topography, composition, and shading. Images were obtained with a resolution of 4 nm, under an accelerating voltage of 0.5 to 20 kV. Furthermore, a FT-600 spectrometer (Agilent Technologies, USA) equipped with an ATR accessory was employed to characterize the functional group on [Nb­(Bez­(COO)_2_)]_*n*_. The mid-infrared range (400 to 4000 cm^–1^) was used to perform the measurements.

### Determination of the Point of Zero Charge (pH_PZC_) of [Nb­(Bez­(COO)_2_)]_*n*_

To study the pH of the point of zero charge (pH_PZC_) of the compound [Nb­(Bez­(COO)_2_)]_*n*_ the methodology described by Vieira et al.^20^ was used, with minor adaptations. Ten samples were prepared in individual vials containing 10.00 mL of 0.1 mol L^–1^ NaCl aqueous solution. The initial pH of each solution was adjusted to values between 2 and 11 using 0.1 mol L^–1^ HCl or 0.1 mol L^–1^ NaOH solutions. Then, 50.00 mg of the compound [Nb­(Bez­(COO)_2_)]_*n*_ were added to each vial, which were kept under mechanical stirring at 40 rpm for 24 h at room temperature. After this period, the final pH of each solution was measured. The pH variation (ΔpH) was then calculated according to eq 1.

1 $$\Delta pH=pH_{Final}-pH_{Initial}$$

### Optimization of Solid Phase Extraction (SPE) of Caffeine

Caffeine extraction was performed based on the SPE technique. This method involves four main steps: (i) preparation of the SPE cartridge containing the synthesized MOF (adsorbent); (ii) conditioning of the MOF; (iii) application of the sample; and finally (iv) elution of the analyte, using an appropriate solvent.

In order to optimize the conditions for caffeine extraction, a multivariate experimental design of type 2^3^ with central compound design (CCD) was conducted. Three independent variables were evaluated: (i) adsorbent mass (100, 250, and 500 g); (ii) pH value of the solution (5.0, 7.4, and 9.0) and (iii) process temperature (25, 35, and 45.0 °C). The variables were coded and organized at different levels, as shown in Table 1.

**Table 1:** Decoded Levels of Variables

variable	symbol	decoded levels		
		–1	0	+1
temperature (°C)	X_1_	25	35	45
pH	X_2_	5.0	7.4	9.0
adsorbent mass (mg)	X_3_	100	250	500

This multivariate approach allows the simultaneous evaluation of individual effects and interactions between factors, enabling the identification of the best experimental conditions to maximize the efficiency of caffeine extraction from the studied material. Table S.1 provides the matrix describing the experimental design.

### Caffeine Quantification

Caffeine extraction was carried out using a manifold system designed for SPE extraction. In each experiment, specific amounts of MOF material (100, 250, or 500 mg) were carefully packed into 5.00 mL polyethylene cartridges, with a layer of glass wool at the base to prevent loss of material during the extraction. Prior to sample loading, the cartridges were conditioned with 5.00 mL of ultrapure water or buffer solution (with pH adjusted to 5.0, 7.4, or 9.0) in different temperatures (25, 35, or 45 °C), considering the Table 1. These conditions allow to activate the MOF surface and increase adsorption reproducibility. Next, 1.00 mL of ta spiked aqueous caffeine solution (10.0 μg mL^–1^) was loaded into the cartridge containing the previously conditioned MOF. The eluted fraction was collected in 15.0 mL Falcon tubes. No additional desorption step was required since the analytical procedure was based on monitoring the decrease in caffeine concentration due to adsorption by the MOF. The collected fraction was filtered through a nylon membrane (0.45 μm, 4 mm, Sartorius, Germany) to remove particles and analyzed using Thermo Scientific Genesys 50 spectrophotometer, at λ_max_ = 275 nm. The efficiency of the extraction process was determined based on the calculation of the percentage yield of the analyte extraction, as expressed in eq 2

2 $$Recovery\left(\%\right)=\left(\frac{Cf}{Ci}\right)$$

Where, c_i_ and c_f_ are initial concentration and concentration of caffeine after extraction, respectively. Calibration curves ranging from 1.0 to 20.0 μg mL^–1^ was used.

### Adsorption Behavior Study

#### Adsorption Isotherm

The experiments related to the adsorption of isotherms were conducted based on the methodology described by de Carvalho et al.,^21^ with minor adaptations. For this purpose, ten caffeine solutions in water were prepared, with concentrations ranging from 2.0 to 10.0 μg mL^–1^, totaling 25.0 mL in each vial. Then, 10.0 mg of the material, [Nb­(Bez­(COO)_2_)]_i_, previously activated with buffer solution (pH 7.4) were added individually to each of the prepared solutions. The samples were kept under constant stirring for a period of 24 h, at the optimum temperature (25 °C), in order to ensure equilibrium between the solid phase and the solution. After the contact time, the systems were subjected to centrifugation at 4,000 rpm, aiming at the separation of the adsorbent solid. The residual caffeine concentration in the supernatant was determined by UV–vis spectrophotometry, at λ_max_ = 275 nm. Calibration curve ranging from 1.0 to 20.0 μg mL^–1^ was used. The amount of caffeine adsorbed at equilibrium (q_e_, in mg g-^1^) was calculated based on eq 3

3 $$q_{e}=\frac{\left(C_{i}-C_{e}\right)xV}{m}$$

Where *Ce* and *Ci* represent, respectively, the equilibrium and initial caffeine concentrations (mg L^–1^). *m* is the volume of the caffeine solution (L) and *m* corresponds to the mass of the adsorbent material used (mg). To evaluate the model that best describes the experimental data obtained in the adsorption isotherms, two well-known mathematical models were applied: (i) Langmuir isotherm (eq 4), (ii) Freundlich isotherm (eq 5), (iii) Temkin isotherm (6); (iv) Redlich-Peterson isotherm.

4 $$q_{e}=\frac{q_{\max}\times K_{L}\times C_{e}}{1+K_{L}\times Ce}$$

5 $$q_{e}=K_{F}\times C_{e}^{1/n}$$

6 $$q_{e}=B\times \ln\left(A\times C_{e}\right)$$

7 $$q_{e}=\frac{K_{R}\times C_{e}}{1+\alpha_{R}\times C_{e}^{\beta }}$$

Where *q*_max_ represents the maximum adsorption capacity, expressed in mg g^–1^, and K_L_ is the Langmuir equilibrium constant, associated with the adsorption energy, with units of L mg^–1^.

K_F_ is the Freundlich equilibrium constant, which reflects the adsorption capacity of the system, with units of mg^1–1/n^ L^1/n^ g^–1^. The variable *n* refers to the heterogeneity index of the adsorbent surface, indicating variations in the energy of active sites. Values of *n* < 1 suggest weaker interactions and greater heterogeneity, while values of 1*n* > 1 indicate stronger interactions and higher affinity between the adsorbate and the adsorbent material.^22^

In the Temkin isotherm model, the constant A (L g^–1^) represents the equilibrium of the system and is directly related to the affinity of the adsorbent for the adsorbate. The parameter B, in turn, is defined by the equation B = RT/b, where R is the universal gas constant (8.314 J mol^–1^ K^–1^), T is the absolute temperature in Kelvin, and b is a constant related to the heat of adsorption (J mol^–1^). This model assumes that the heat of adsorption of all molecules in the layer decreases linearly with surface coverage, reflecting interactions between the adsorbate and the adsorbent.

In the Redlich–Peterson model, the constant K_R_ (L g^–1^) represents the Redlich–Peterson constant related to the adsorption capacity of the system. The constant α_R_ ((L mg^–1^)^β^) is associated with the adsorption energy. Finally, the parameter β is a dimensionless exponent characteristic of the Redlich–Peterson model, ranging between 0 and 1, and it reflects the heterogeneity of the adsorbent surface.

#### Adsorption Kinetic

The adsorption kinetics study was conducted based on the methodology proposed by de Jesus,^23^ with minor adaptations. In this procedure, 10.0 mg of [Nb­(Bez­(COO)_2_)]_*n*_, previously activated with buffer solution (pH 7.4), were added to a beaker containing 25.0 mL of a caffeine solution at a concentration of 10 μg L^–1^, maintained under constant agitation at 250 rpm. At predetermined time intervals, aliquots were withdrawn to determine the amount of adsorbed caffeine (q_t_, in mg g^–1^) using UV–vis spectrophotometry, at λ_max_ = 275 nm according to eq 8. Calibration curve ranging from 1.0 to 18.0 μg mL^–1^ was used. After each analysis, the aliquots were returned to the system to maintain the solution volume and consistency throughout the experiment

8 $$q_{t}=\frac{\left(C_{i}-C_{e}\right)xV}{m}$$

Where q_t_ is the amount of caffeine at specific time, *Ce* and *Ci* represent, respectively, the equilibrium and initial caffeine concentrations (mg L^–1^). *V* is the volume of the caffeine solution (L) and *m* corresponds to the mass of the adsorbent material used (mg). To evaluate the model that best describes the experimental data obtained in the kinetic adsorption of caffeine onto the material, pseudo-first-order model (eq 9), pseudo-second-order model (eq 10) and intraparticle diffusion model (eq 11) were used.

9 $$\ln\left(q-q_{t}\right)=\ln q_{e}-K_{1}t$$

10 $$\frac{t}{q_{t}}=\frac{1}{{\left(K_{2\cdot }t\right)}^{2}}+\frac{t}{q_{e}}$$

11 $$q_{t}=k_{id}\times t^{1/2}+C$$

where *K*_1_ and *K*_2_ are adsorption rate constants, expressed in g mg^–1^ min^–1^. The parameter *k*_id_ represents the intraparticle diffusion rate constant (mg g^–1^ min^–0^·^5^), which reflects the rate at which the adsorbate migrates into the internal pores of the adsorbent material. The constant C (mg g^–1^) is associated with the thickness of the boundary layer surrounding the adsorbent particles. When C ≈ 0, it indicates that the adsorption process is primarily governed by intraparticle diffusion. In contrast, values of C > 0 suggest a significant influence of external mass transfer resistance, implying that multiple kinetic steps are involved in the overall adsorption mechanism.

#### Thermodynamic Study

To evaluate the thermodynamic properties of the caffeine with [Nb­(Bez­(COO)_2_)]_*n*_, a study was conducted at three temperatures (281, 298, and 315 K), following the methodology adapted from Silva et al.^24^ In this procedure, 10.0 mg of the adsorbent was added to 10.0 mL of diluted caffeine solution at a concentration of 13 μg mL^–1^. The mixture was subjected to continuous agitation at 40 rpm for 24 h while maintained at constant temperature. After this period, the solutions were centrifuged at 4,000 rpm, and the concentration of caffeine remaining in the supernatant was determined by measuring absorbance at the maximum wavelength λ_max_ = 275 nm. Calibration curve ranging from 5.0 to 20.0 μg mL^–1^ was used.

The amount of caffeine adsorbed at equilibrium (q_e_, mg g^–1^) was calculated using eq 3. Thermodynamic parameters such as changes in Gibbs free energy (Δ*G*°, kJ mol^–1^), enthalpy (Δ*H*°, kJ mol^–1^), and entropy (Δ*S*°, kJ mol^–1^ K^–1^) were subsequently determined. Initially, the distribution coefficient (K_D_) was calculated using eq 12. The Gibbs free energy change (Δ*G*°) was obtained at each temperature (281, 298, and 315 K) using eq 13. To determine Δ*H*° and Δ*S*°, a plot of the natural logarithm of the equilibrium constant (ln *K*_D_) versus the inverse of absolute temperature (1/T) was constructed. According to the van’t Hoff equation (eq 14), the slope and intercept of the resulting linear fit provided the values of Δ*H*° and Δ*S*°, respectively, characterizing the thermodynamic behavior of the adsorption process.

12 $$K_{D}=\frac{q_{e}}{C_{e}}$$

13 $$\Delta G^{{}^{\circ}}=RT\ln\left(K_{D}\right)$$

14 $$\ln K=\frac{-\Delta H}{R}\left(\frac{1}{T}\right)+\frac{\Delta S}{R}$$

In these equations, *R* is the universal gas constant (8.314 J K^–1^ mol^–1^), *T* represents the absolute temperature in Kelvin, and *K*_D_ corresponds to the distribution coefficient at equilibrium.

#### Figures of Merit

To assess the applicability of the developed method, several analytical parameters were evaluated, including selectivity, accuracy, precision, limit of detection (LOD), and limit of quantification (LOQ).^17^ Calibration curve was constructed using standard solutions with concentrations ranging from 1.0 to 20.0 μg mL^–1^. Accuracy was determined based on spike experiment. Recovery rates (%) were calculated according to eq 2, while method precision was assessed by determining the relative standard deviation (RSD). The LOD and LOQ values were estimated using Eq. 15 and ([Disp-formula eq16]), respectively

15 $$LOD=3x\left(\frac{S_{b}}{m}\right)$$

16 $$LOQ=10x\left(\frac{S_{b}}{m}\right)$$

In these equations, *S*_*b*_ is the standard deviation of the blank measurements (*n* = 10), a sample free of caffeine, while *m* represents the slope obtained from the analytical calibration curve.

#### Statistical Analysis

The multifactorial analysis data was processed using STATISTICAL software (version 7). Analysis of Variance (ANOVA) was conducted to identify significant variables, considering a significance threshold of *p* < 0.05.

## Results and Discussion

### Characterization of [Nb­(Bez­(COO)_2_)]_*n*_

Several structural characterization techniques were applied to the synthesized material, [Nb­(Bez­(COO)_2_)]_*n*_. The FT-IR spectrum obtained for the MOF (Figure 2A) synthesized from Bez­(COO)_2_ (Figure 2B) and Nb revealed characteristic bands that confirm the coordination between the organic ligand and the metal. A broad band between 3200 and 3500 cm^–1^ is observed, attributed to the stretching vibrations of the hydroxyl group (−OH), which may indicate the presence of coordinated water molecules or water of hydration in the material. The intense bands observed around 1680–1720 cm^–1^ are attributed to the stretching vibration of the carbonyl group (C=O) of the carboxylic groups of terephthalic acid. However, after coordination with Nb, these bands shift to lower frequencies (1540–1600 cm^–1^ and 1380–1420 cm^–1^), indicating the formation of Nb–O–C bonds between the metal and the carboxylic ligand. This shift is a typical indication of deprotonation of carboxylic acid and subsequent coordination to the metal center. Additionally, additional bands in the region of 500–800 cm^–1^ can be attributed to Nb–O stretching vibrations, confirming the presence of the metal in the framework.

**Figure 2:**
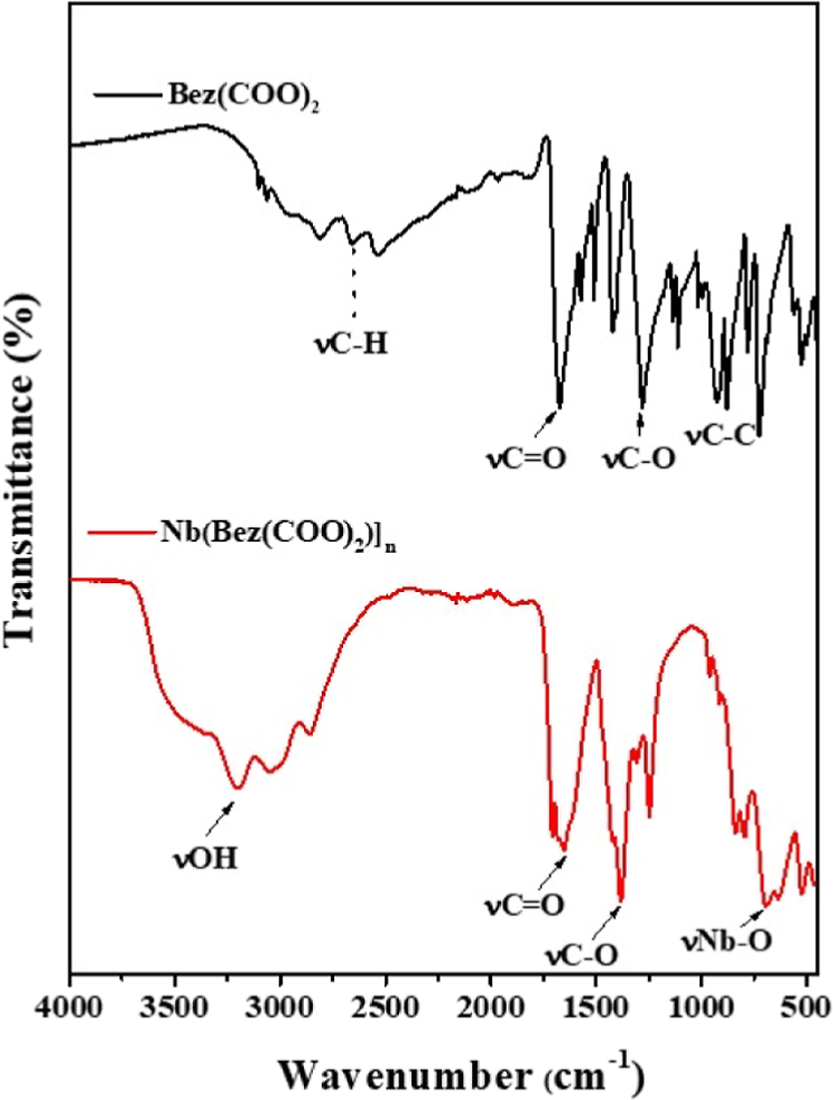
Infrared spectra of the synthesized material [Nb­(Bez­(COO)_2_)]_*n*_, and the ligand 1,4-benzenedicarboxylic acid (Bez­(COO)_2_).

Figure 3 presents the Raman spectroscopy characterization, where the most prominent peaks were identified in the ranges of 600–700 cm^–1^, 1200–1500 cm^–1^, and 200–300 cm^–1^, which are typically associated with the vibrational modes of Nb–O bonds.^25,26^ Peaks between 900 and 1140 cm^–1^ correspond to the presence of the ligand into material.^27,28^

**Figure 3:**
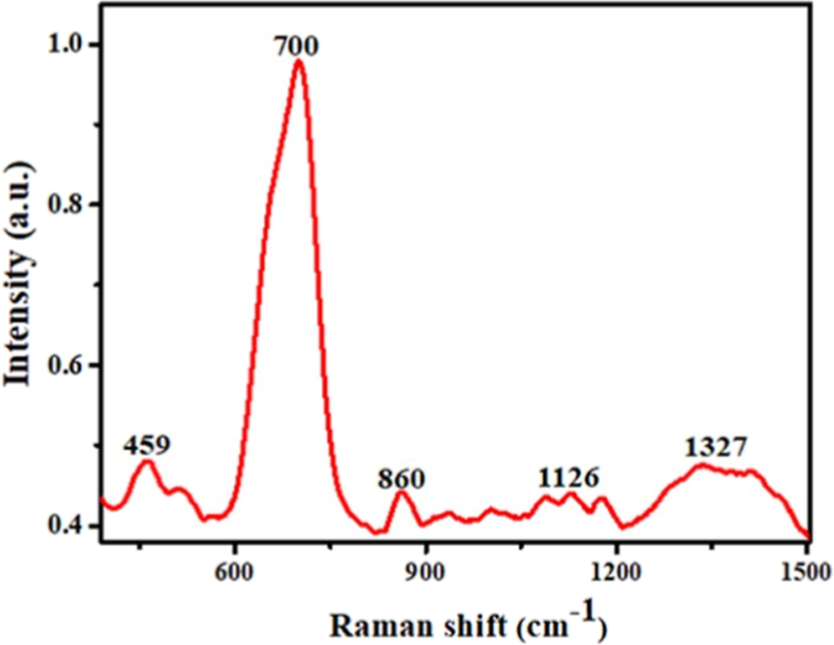
Raman spectrum of the synthesized material, [Nb­(Bez­(COO)_2_)]_*n*_.

These results together of FT-IR and Raman spectroscopy demonstrate that Bez­(COO)_2_ was effectively incorporated into the material and coordinated to Nb, forming a stable MOF.

The morphological analysis of the material [Nb­(Bez­(COO)_2_)]_*n*_ by SEM (Figure 4A) revealed a structure predominantly formed by rods with well-defined hexagonal geometry. This morphology is characteristic of highly organized systems, suggesting that the synthesis and crystallization conditions employed successfully promoted anisotropic crystal growth.^29–31^ The formation of hexagonal rods can be attributed to the coordinated organization between Nb and the ligand, reflecting a strong interaction between the metal center and the carboxylate groups of the ligand. Furthermore, EDS analysis (Figure 4B), confirmed the presence of Nb in the material, as evidenced by the characteristics elemental peaks observed.

**Figure 4:**
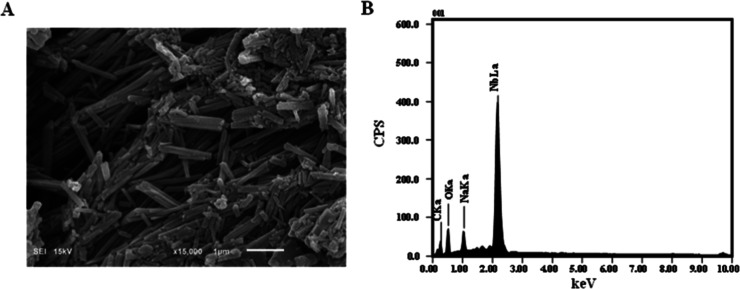
(A) Scanning Electron Microscopy (SEM) micrograph, and (B) Energy-Dispersive X-ray Spectroscopy (EDS) analysis of [Nb­(Bez­(COO)_2_)]_*n*_.

Furthermore, the XRD diffractogram (Figure 5A) presents features indicative of high crystallinity, corroborated by the presence of flat surfaces and well-defined angles observed in the micrographs (Figure 5B), characteristic of a monoclinic crystal system. These structural properties are in good agreement with the crystallographic standard (ICDD 01–074-2447), suggesting the existence of well-defined crystallographic planes within the [Nb­(Bez­(COO)_2_)]_*n*_ structure. The calculated crystallite size was approximately 6.65 nm. These findings corroborate other structural characterization results, such as SEM analysis. Thus, the observed morphology not only confirms the successful synthesis of the targeted material but also suggests the presence of specific physicochemical properties associated with its high crystallinity and organized framework.

**Figure 5:**
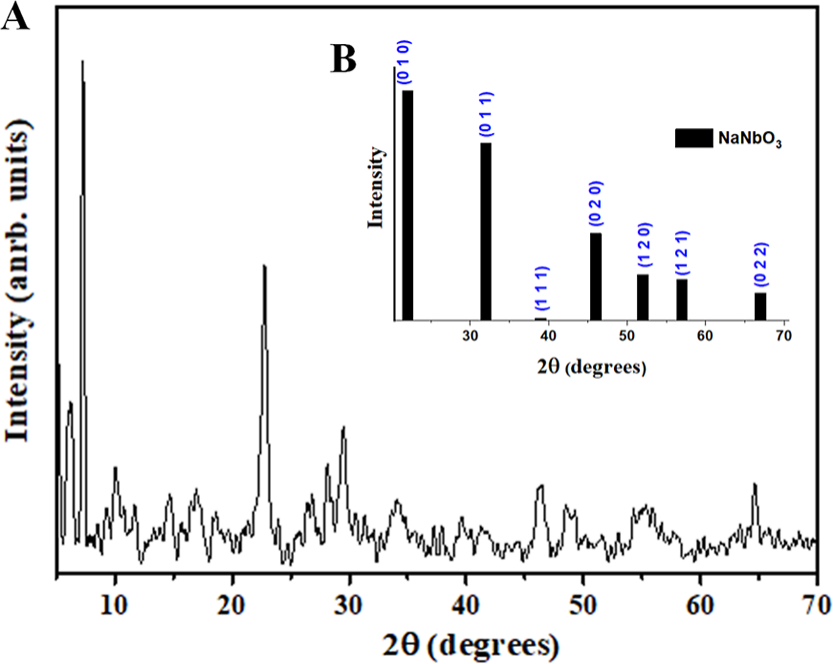
(A) X-ray diffractogram of [Nb­(Bez­(COO)_2_)]_*n*_. (B) Crystallographic pattern of NaNbO_3_ (ICDD 01-074-2447), showing similarity to the experimental data for [Nb­(Bez­(COO)_2_)]_*n*_ and suggesting possible crystallographic planes in the synthesized material.

From the BET analysis (Figure 6A), the N_2_ adsorption–desorption curve exhibits the profile of a type IV isotherm, indicating multilayer adsorption, which is characteristic behavior of mesoporous structures.^32^ In addition, the presence of type H1 hysteresis loop was observed, indicating existence of well-defined pores on the material’s surface. The specific surface area was found to be 94.7063 m^2^ g^–1^, indicating that the material is suitable for applications that require high surface area and porosity, such as the adsorption of analytes, thereby confirming the suitability of the synthesized material for such applications, [Nb­(Bez­(COO)_2_)]_*n*_.^19^

**Figure 6:**
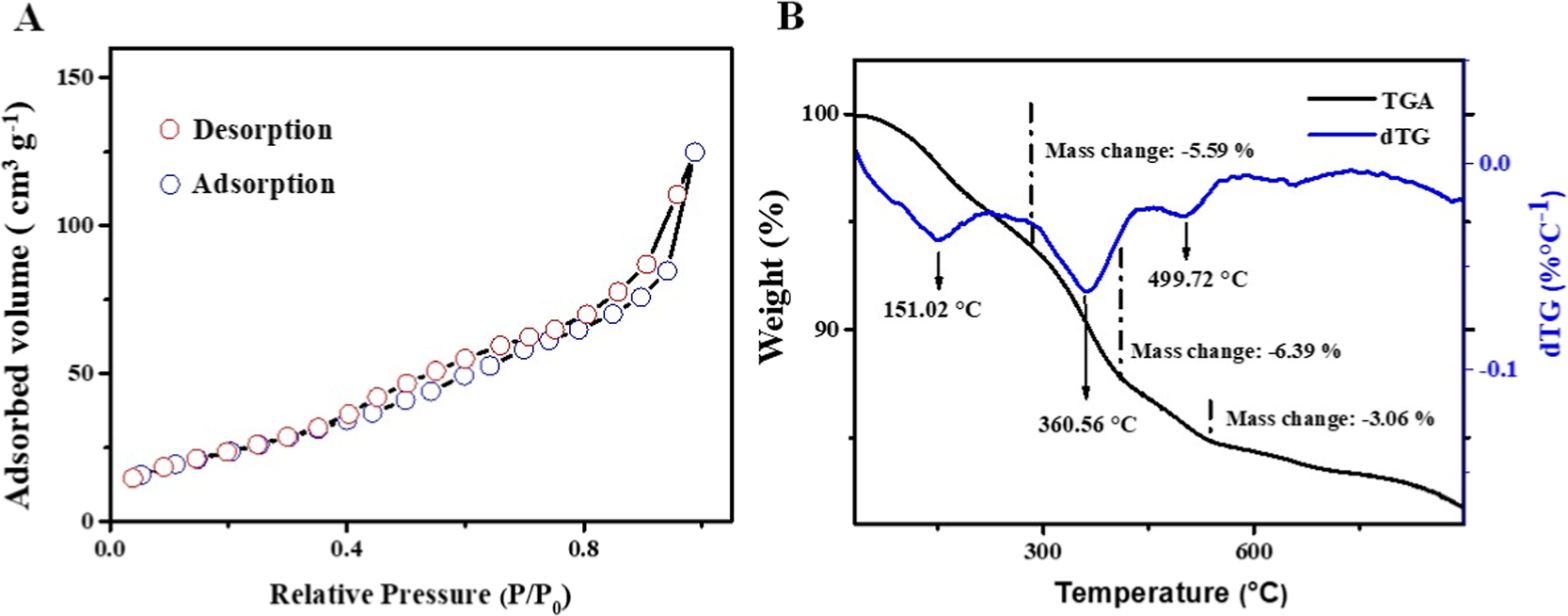
(A) Brunauer–Emmett–Teller (BET) surface area analysis, and (B) thermogravimetric analysis (TGA) curve of [Nb­(Bez­(COO)_2_)]_*n*_.

The TGA analysis (Figure 6B) reveals three distinct thermal events. The first event, characterized by a 5.59% mass loss at 151.2 °C, is attributed to the evaporation of residual organic solvent trapped within the material. The second event, observed at 360.56 °C with a mass loss of 6.39%, corresponds to the onset of thermal degradation of the Bez­(COO)_2_ ligand.^19,33^ Finally, the third event, marked by a 3.06% mass loss at 499.72 °C, indicates the complete decomposition of the Bez­(COO)_2_ ligand.^16^ These thermal behaviors corroborate the structural integrity and composition of the synthesized material.

Through the investigation of the pH_PZC_, it was observed that the material exhibits a PZC at pH 11 (Figure 7). At this specific pH, the overall balance of negative and positive charges on the nanomaterial surface is equal. Consequently, the surface acquires a negative charge at pH values above 11 and a positive charge at values below 11. By comparing this PZC value of 11.0 with the p*K*_a_ of caffeine (8.3), as reported in the literature,^34^ it becomes evident that the behavior of the MOF at this pH can favor the adsorption of the analyte.

**Figure 7:**
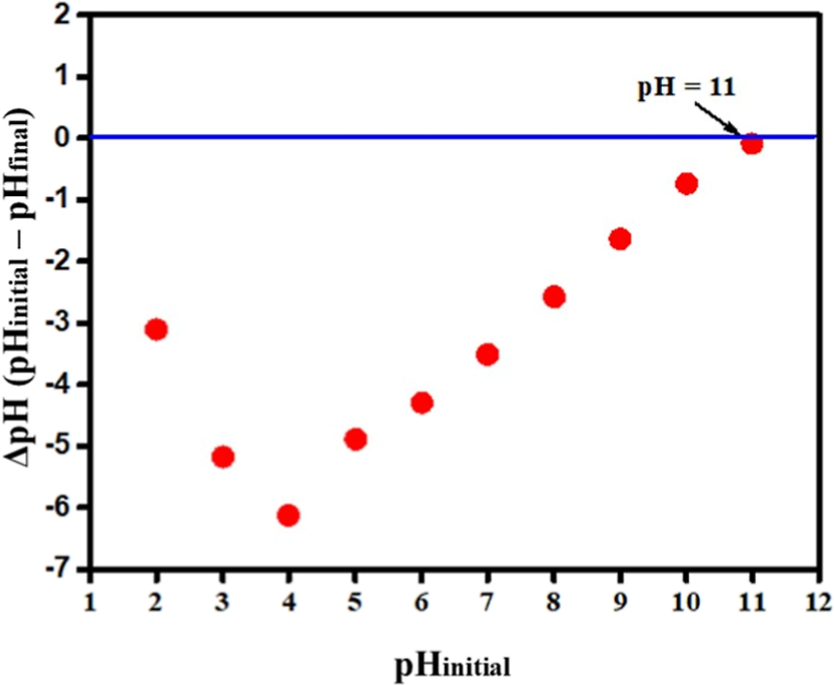
Determination of the point of zero charge (PZC) of [Nb­(Bez­(COO)_2_)]_*n*_.

### Multivariate Optimization Employing [Nb­(Bez­(COO)_2_)]_*n*_

It is widely recognized that solid-phase extraction (SPE) performance can be significantly influenced by multiple factors.^35^ In this work, three critical factors were evaluated using a multivariate approach: (i) the MOF mass (10.0, 25.0, and 50.0 mg), (ii) the solution pH (5.0, 7.4, and 9.0), and (iii) the extraction temperature (25, 35, and 45 °C). A 2^3^ full factorial design was initially implemented to evaluate both the main effects and the interactions of two factors, with the experimental matrix detailed in Table S.1. To further explore potential nonlinear relationships and optimize the extraction conditions, a central composite design (CCD) was subsequently employed. This strategy, coupled with the response surface methodology (RSM), allowed the modeling of the system behavior with greater accuracy and the identification of optimal operating parameters.

Table S2 presents the results obtained after caffeine extraction using a multifactor optimization approach, while Figure S1 illustrates the corresponding Pareto diagram. Analysis of Figure S1 reveals that adsorbent mass, pH, and their interaction were the statistically significant variables at a 95% confidence level. Among these, MOF mass was the most influential factor, strongly impacting the extraction efficiency. Temperature alone was not significant, but when interacted with the other factors, it demonstrated an influence on the extraction process. Notably, the most evaluated factors exhibited a negative effect on the extraction process, suggesting that as the levels of adsorbent mass, pH, or their combination increased, a decrease in caffeine extraction efficiency was observed. This behavior suggests that a greater mass of the adsorbent may lead to aggregation or saturation phenomena, reducing the availability of active sites for adsorption, while high pH values may potentially affect the protonation state of the caffeine molecules or the surface charge of the adsorbent, impairing the interaction between the analyte and the material. Based on the Table S2, the response surfaces were built, as presented in Figure 8. The optimal conditions for caffeine extraction were determined tobe (i) 100 mg of [Nb­(Bez­(COO)_2_)]_*n*_, (ii) pH 9.2, and (iii) temperature of 25 °C.

**Figure 8:**
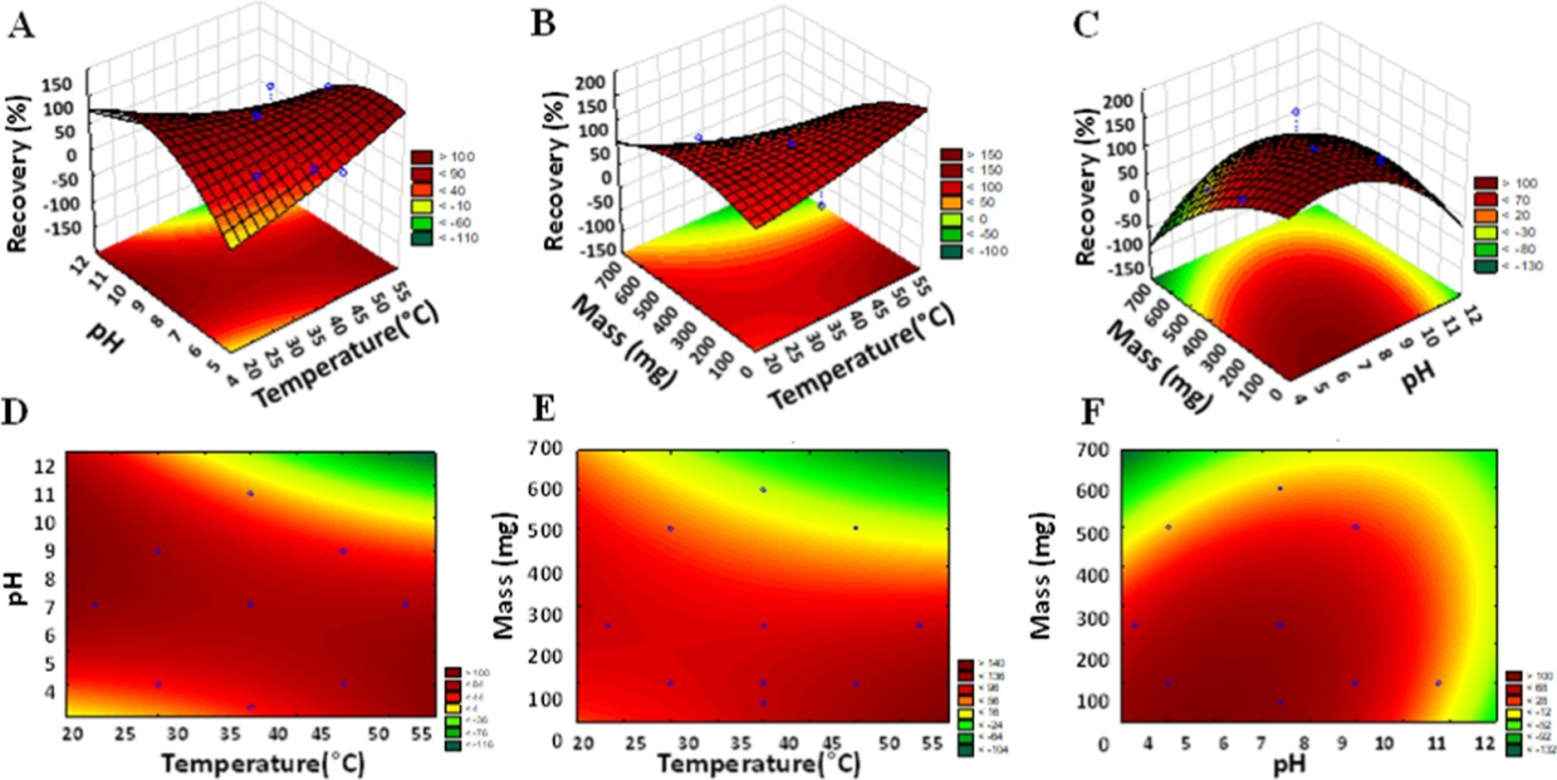
Response surface generated through multivariate optimization for caffeine extraction using [Nb­(Bez­(COO)_2_)]_*n*_. Panels A, B, and C represent the three-dimensional response surfaces, while panels D, E, and F correspond to the level curves.

The results of the analysis of variance (ANOVA), summarized in Table 2, revealed that the mass of [Nb­(Bez­(COO)_2_)]_*n*_, as well as the three two-way interactions between the variables, were statistically significant factors influencing the extraction efficiency, at a 95% confidence level (*p* < 0.05). In contrast, the effect of temperature variation alone was not statistically significant under the conditions tested. These findings highlight the critical role of sorbent mass and interactive effects, while suggesting that, within the evaluated range, temperature exerts a negligible direct impact on the extraction process. The model exhibited an *R* ^2^ value of 0.98, demonstrating a strong correlation and a high degree of data adjustment to the proposed model.

**Table 2:** Central Compound Delineation (CCD) parameters^a^

factor	sum quadratic (S)	degrees of freedom	*F*_calc_	p
X_1_	31.62	1	0.4532	>0.05
X_2_	35.32	1	0.5063	<0.05
X_3_	9426.48	1	135.1017	<0.05
interaction x_1_ vs x_2_	1987.55	1	28.4858	<0.05
interaction x_1_ vs x_3_	1807.68	1	25.908	<0.05
interaction x_2_ vs x_3_	1804.14	6	25.8572	<0.05
pure error	418.64			
residue	69.7732			
*R* ^2^	0.983			
*R*-adjusted	0.959			

a A variável x_1_ corresponde a temperature, x_2_ é o pH, x_3_ é a massa de [Nb­(Bez­(COO)_2_)]_*n*_.

Figure S2 shows the model adjustment, illustrating the correlation between the predicted and experimental values, thus confirming the robustness of the model and the efficiency of the method for caffeine extraction from the sample.

### Adsorption Isotherms Employing [Nb­(Bez­(COO)_2_)]_*n*_

The experimental data on caffeine adsorption onto the [Nb­(Bez­(COO)_2_)]_*n*_ were analyzed using the Langmuir and Freundlich models to investigate the adsorption mechanism and evaluate the material’s adsorption efficiency. The results from the model fittings are presented in Figure S3 and Table 3.

**Table 3:** Parameters of Isotherm Models

	parameters	caffeine
Langmuir	*R* ^2^	0.8907
	*q*_max_ (mg g^–1^)	14.7437
	K_L_ (mg^–1^)	0.0359
Freundlich	*R* ^2^	0.9498
	K_F_ (mg^(1–1/n)^ L^1/n^ g^–1^)	4.952
	n	4.766
Temkin	*R* ^2^	0.8024
	A (L g^–1^)	0.3459
	B	3.1899
Redlich-Peterson	*R* ^2^	0.8435
	K_R_	0.4171
	α_R_	5.56 × 10^–11^
	g	8.3745

For caffeine, the Freundlich model provided a superior fit to the experimental data, with an *R* ^2^ value of 0.9498, compared to 0.8907 for the Langmuir model, 0.8024 for the Temkin model and 0.8435 for the model Redlich-Peterson. This better fit suggests a heterogeneous surface with adsorption sites of varying energies, in contrast to the uniform monolayer adsorption assumed by the Langmuir model.^36^ The Freundlich constant (K_f_) was determined to be 4.952 mg^(1–1/n)^ L^1/n^ g^–1^, indicating favorable adsorption and significant interactions between caffeine molecules and the surface of [Nb­(Bez­(COO)_2_)]_*n*_.^36–38^ Furthermore, the Freundlich exponent (*n*) was calculated to be 4.766, which is greater than 1, confirming the favorable nature of the adsorption process. These results highlight the high efficiency of [Nb­(Bez­(COO)_2_)]_*n*_ in removing caffeine from aqueous solutions, probably due to the presence of multiple active sites on the surface of the material. In contrast, the lower correlation observed for the Langmuir model suggests that uniform monolayer adsorption is less representative of the studied system. Although the maximum adsorption capacity (*q*_max_) estimated by the Langmuir model provides a theoretical limit for adsorption, it does not adequately capture the experimentally observed surface heterogeneity.

The superior fit to the Freundlich model suggests that interactions such as van der Waals forces, hydrogen bonding, or π–π interactions between caffeine molecules and the [Nb­(Bez­(COO)_2_)]_*n*_ may play a significant role in the adsorption process.^39^ These findings reinforce that the caffeine adsorption process on [Nb­(Bez­(COO)_2_)]_*n*_ is more consistent with a heterogeneous energy surface, resulting in different adsorption interactions.

### Adsorption Kinetic

The interaction mechanism between the [Nb­(Bez­(COO)_2_)]_*n*_ and caffeine, along with the rate-determining steps in the adsorption process, was explored by applying kinetic models. The adsorption kinetic data obtained were then fitted using the pseudo-first-order, pseudo-second-order and intraparticle diffusion models. The adsorption kinetics of caffeine using [Nb­(Bez­(COO)_2_)]_*n*_ as the adsorbent were evaluated by fitting the experimental data to the pseudo-first-order (Figure 9A), pseudo-second-order models (Figure 9B) and intraparticle diffusion (Figure 9C).

**Figure 9:**
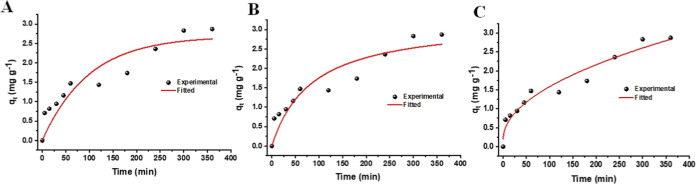
Caffeine adsorption kinetics fitted to the (A) pseudo-first-order model; (B) pseudo-second-order model; (C) intraparticle diffusion model.

As shown in Figure 9, the caffeine adsorption process exhibits a rapid initial absorption followed by a gradual progression toward equilibrium in approximately 400 min. This biphasic behavior suggests that multiple steps may govern the adsorption kinetics. According to the data in Table 4, the pseudo-second-order model presents a fit with *R* ^2^ = 0.8641, while the pseudo-first-order model showed a fit with *R* ^2^ = 0.8247. This suggests that the adsorption mechanism may involve significant surface interactions consistent with physisorption, such as hydrogen bonding and van der Waals forces between the caffeine molecules and [Nb­(Bez­(COO)_2_)]_*n*_.

**Table 4:** Parameters of Kinetic Models

	parameters	caffeine
Pseudo-first-order	q_e_ (mg g^–1^)	2.6904
	K (1/min)	0.0101
	*R* ^2^	0.8247
Pseudo-second-order	q_e_ (mg g^–1^)	3.2414
	K (1/min)	0.00364
	*R* ^2^	0.8641
intraparticle diffusion	C (mg g^–1^)	0.19228
	K (mg g^–1^ min^–0·5^)	0.13924
	*R* ^2^	0.9554

To gain a deeper understanding of the mass transfer mechanism, the intraparticle diffusion model was applied. The resulting correlation coefficient was remarkably high (*R* ^2^ = 0.9554), indicating that this model describes the experimental data more accurately than the other kinetic models (Table 4). The obtained parameters, *k*_id_ = 0.1392 mg g^–1^ min^–0.5^ and *C* = 0.1923 mg g^–1^, suggest that although intraparticle diffusion plays a significant role, it is not the only rate-limiting step. The nonzero value of *C* points to the additional influence of external resistance to mass transfer in the early stages of adsorption. Together, these results confirm that caffeine adsorption on [Nb­(Bez­(COO)_2_)]_*n*_ occurs through a combination of surface interaction and intraparticle diffusion, which is consistent with the heterogeneous surface characteristics of the material and its moderate specific surface area.

### Thermodynamic Study

The thermodynamic study of caffeine adsorption onto the [Nb­(Bez­(COO)_2_)]_*n*_ was conducted at temperatures of 298, 308, and 323 K (Figure S4) to evaluate the spontaneity, energy changes, and nature of the adsorption process. The Gibbs free energy (Δ*G*) values for these temperatures were −5.052, −5.008, and −4.668 kJ mol^–1^, respectively, with a high correlation coefficient (*R* ^2^ = 0.97) (Table 5).

**Table 5:** Thermodynamic Model Parameters

caffeine	Δ*H*° (kJ mol^–1^)	Δ*S*° (J mol^–1^ K^–1^)	Δ*G*° (kJ mol^–1^)			*R* ^2^
			298 K	308 K	323 K	
	–3026	–0.0156	–5.052	–5.008	–4.668	0.97

The negative Δ*G* values at all tested temperatures confirm that the adsorption of caffeine onto the [Nb­(Bez­(COO)_2_)]_*n*_ is a spontaneous process. The decrease in the magnitude of Δ*G* with increasing temperature indicates a reduction in spontaneity as temperature rises. This finding corroborates the ANOVA result (Table 2) for the caffeine extraction experiment, which indicates that temperature is not a significant variable. This trend suggests that the adsorption process is exothermic, where higher temperatures slightly reduce the extent of adsorption by shifting the equilibrium toward desorption.

Furthermore, the exothermic nature of the process is consistent with the observed thermodynamic behavior and aligns with the strong material affinity for caffeine molecules at lower temperatures. This behavior could be attributed to specific interactions, such as hydrogen bonding or π–π stacking, which are energetically favorable but may weaken with increased thermal agitation at higher temperatures.

On the other hand, the observed negative entropy change (Δ*S*° = −0.0156 kJ mol^–1^ K^–1^) indicates a decrease in randomness at the solid–liquid interface during adsorption, consistent with adsorbate molecules becoming more ordered upon binding to the MOF surface. Although physisorption is often considered entropically driven due to solvent displacement and increased disorder in the bulk phase, adsorption in porous materials like MOFs can lead to confinement of adsorbate molecules within well-defined pores or specific sites. This spatial restriction reduces the translational and rotational freedom of adsorbates, resulting in a net decrease in entropy.^40^ Several studies have reported similar thermodynamic behavior in MOFs and related porous materials, where enthalpy-driven adsorption is accompanied by negative entropy changes due to adsorbate ordering and pore confinement^41^

Thus, the thermodynamic data suggest spontaneous adsorption primarily driven by enthalpic interactions (e.g., van der Waals forces, hydrogen bonding) with a compensating entropy decrease caused by adsorbate immobilization in the porous structure, consistent with the observed low enthalpy change and physisorption characteristics.

These results provide further evidence that caffeine adsorption onto the MOF is favorable under the studied conditions. However, the gradual reduction in spontaneity with rising temperature underscores the importance of optimizing adsorption processes for practical applications, particularly when considering thermal variations.^42,43^

### Potential Mechanism of Interaction Between Caffeine and [Nb­(Bez­(COO)_2_)]_*n*_

The adsorption mechanism of caffeine on [Nb­(Bez­(COO)_2_)]_*n*_ is predominantly governed by physisorption coupled with intraparticle diffusion, as evidenced by the good fit of the experimental data to the intraparticle diffusion kinetic model. Under the experimental conditions (pH 9.0) and considering the zero charge point of the material (pH_PZC_) of 11, the surface of [Nb­(Bez­(COO)_2_)]_*n*_ is positively charged.

Caffeine, with a p*K*_a_ of approximately 8.2, exists predominantly in the negatively charged form at pH 9.0 due to deprotonation of functional groups, increasing the polarity of the molecule. These changes may promote electrostatic interactions between partially negative regions of caffeine, such as the carbonyl and nitrogen groups, and the positively charged surface of the MOF (since pH 9.0 < PZC 11). Therefore, electrostatic attraction may contribute to the overall adsorption mechanism.

Furthermore, van der Waals forces, transient-induced dipole–dipole interactions, contribute significantly to adsorption, stabilizing caffeine molecules on the MOF surface. The overall process is characteristic of physisorption. On the other words, it is reversible, occurs at low activation energy, and does not involve the formation of strong chemical bonds. Although, hydrogen bonds can also occur.^44,45^

The heterogeneous nature of the [Nb­(Bez­(COO)_2_)]_*n*_ surface, provides a variety of adsorption sites with different affinities, increasing the material efficiency in capturing caffeine molecules. Figure S5 presents the FT-IR spectra of pure caffeine and caffeine adsorbed on [Nb­(Bez­(COO)_2_)]_*n*_, highlighting the efficiency of the material in capturing caffeine. The comparison between the spectra reveals subtle changes in the position and intensity of specific bands, particularly in the regions associated with carboxylate functional groups and vibrations of the aromatic ring. These spectral changes may indicate the occurrence of noncovalent interactions between the caffeine molecules and the active sites of the material. The preservation of the characteristic bands of the Nb–O network also indicates that the structure of the material remained stable after the adsorption process, confirming its structural robustness.

Therefore, adsorption is primarily governed by a combination of electrostatic attractions and van der Waals forces, confirming the suitability of [Nb­(Bez­(COO)_2_)]_*n*_ as an efficient adsorbent for the removal of emerging contaminants such as caffeine via a predominantly physical adsorption mechanism.^44,45^

### Figures of Merit

After developing the method for the extraction of caffeine using the material [Nb­(Bez­(COO)_2_)]_*n*_, essential analytical parameters such as linear range, linearity, LOD and LOQ were evaluated. The method presented a linear range of 1.0 to 20.0 μg mL^–1^, with excellent linearity, evidenced by a coefficient of determination (*R* ^2^) of 0.9978. The LOD was 0.54 μg mL^–1^ and the LOQ was 1.78 μg mL^–1^, demonstrating good sensitivity for the detection of caffeine in aqueous samples. The accuracy of the method was evaluated using a concentration of 4.53 μg mL^–1^, resulting in a recovery of 95.6 ± 1.1% (*n* = 3) and an absolute error of −0.20 μg mL^–1^. These results confirm the reliability, accuracy and efficiency of the developed SPE method, highlighting the potential of [Nb­(Bez­(COO)_2_)]_*n*_ for analytical applications in the determination of caffeine.

### Reusability Studies

The stability and extraction efficiency of the material [Nb­(Bez­(COO)_2_)]_*n*_ were evaluated with a focus on its reuse. For this purpose, a recovery study was carried out in five consecutive caffeine adsorption/desorption cycles, using the same batch of [Nb­(Bez­(COO)_2_)]_*n*_. Each cycle was conducted under ultrasonic agitation for 15 min. After the desorption process, the material was washed extensively with ethanol, followed by drying at 40 °C for 3 h, before being reused. The results, illustrated in Table 6, indicated satisfactory recoveries up to the fifth use cycle, evidencing the good stability and reusability of the material.

**Table 6:** Efficiency of Reuse of [Nb­(Bez­(COO)_2_)]_*n*_ for Caffeine Extraction (*n* = 3)

cycle	add (μg mL^–1^)	found (±SD μg mL ^–1^)	recovery (±RSD %)
**1**	8.0	9.3 ± 0.01	116 ± 1
**2**	8.0	8.1 ± 0.02	101 ± 3
**3**	8.0	7.8 ± 0.03	97 ± 4
**4**	8.0	6.5 ± 0.05	82 ± 8
**5**	8.0	5.6 ± 0.07	71 ± 12

These data reinforce the potential of [Nb­(Bez­(COO)_2_)]_*n*_ as a sustainable and reusable adsorbent, aligned with the principles of the circular economy and environmentally responsible processes.

### Caffeine Determination in Real Sample

Surface water samples collected from the lagoon of the Federal University of Viçosa were analyzed for the presence of caffeine residues using the proposed method. The analytical results indicated that the caffeine concentrations in the samples were below the LOD of the method.

### Comparison with Other Methods

To demonstrate the performance of the SPE method using [Nb­(Bez­(COO)_2_)]_*n*_, a comparison was made with literature data reporting alternative extraction methods for other analytes. The results of this comparative study are presented in Table 7.

**Table 7:** Comparison of SPE Methods for Contaminants Extraction Using MOFs

technique	extraction sorbent	analyte	sample	linearity range	sample recovery (%)	ref
HPLC-PDA	MOF@RGO	caffeine	exhaled breath condensate	10–500 μg L–^1^	86	^46^
HPLC-UV	MOF NiCoZn-LDH@GO	7 drugs	biological fluid	0.3–1000 ng L–^1^	87.4	^47^
coated blade spray-MS (CBS-MS)	Cu-TCPP/Ti_3_C_2_Tx	21 drugs	environmental water	0.05–10 ng mL^–1^	68–133	^48^
LC–MS/MS	MPN@COFs	caffeine	tea	0.01–1.2 μg L–^1^	79	^49^
UV Spectroscopy	[Nb(Bez(COO)_2_)]_*n*_	caffeine	surface water	1–20 μg mL^–1^	95.6	this work

Abbreviations: CBS-MS: coated blade spray coupled to mass spectrometry; Cu-TCPP/Ti_3_C_2_Tx: porphyrin-based metal–organic frameworks modified with Ti_3_C_2_Tx sheets; HPLC-PDA: high performance liquid chromatography with diode array detector; HPLC-UV: high performance liquid chromatography with ultraviolet detection; LC–MS/MS: liquid chromatography coupled to tandem mass spectrometry; MOF@RGO: cobalt-based metal–organic framework combined with reduced graphene oxide; MPN@COFs: metal-phenolic networks encapsulated in covalent organic frameworks; ZIF-67: zeolitic imidazolate framework-67; MOF NiCoZn-LDH@GO: Metal–Organic Framework based on a Nickel–Cobalt–Zinc Layered Double Hydroxide supported on Graphene Oxide.

From Table 7, it can be observed that the proposed method presents competitive performance, combining high sensitivity (LOD of 0.54 μg mL^–1^) and good recovery (95.6 ± 1.1%). In addition, the material presented high stability and the possibility of reusing for up to five cycles, which reinforces its potential as a more sustainable and efficient alternative for the extraction of caffeine from aqueous samples.

## Conclusions

In this study, an SPE method was optimized using the material [Nb­(Bez­(COO)_2_)]_*n*_ for the removal of caffeine from aqueous solutions. The characterization of the material confirmed its porous structure and high surface area, essential for adsorption performance. The extraction process was optimized by response surface methodology, identifying that the adsorbent mass, solution pH and temperature significantly influence the efficiency of the system, with the optimal extraction conditions being (i) 10 mg of material, (ii) pH 9.0 and (iii) temperature of 25 °C. Isotherm studies revealed that the Freundlich model best describes the adsorption behavior, indicating a heterogeneous surface with multiple active sites. The adsorption kinetics followed the pseudo-second order model, suggesting predominance of physisorption processes. The thermodynamic evaluation indicated that the adsorption is spontaneous and exothermic. The analytical application of the method showed good linearity (*R* ^2^ = 0.9978), adequate detection and quantification limits, and high recovery, proving the effectiveness and sensitivity of the developed system. In addition, the material showed high stability, being successfully reused in at least five adsorption–desorption cycles, without significant loss of efficiency. Thus, [Nb­(Bez­(COO)_2_)]_*n*_ proves to be a promising, sustainable and efficient alternative for the detection and removal of caffeine in aqueous matrices, contributing to environmental monitoring.

## Supplementary Material



## Notes

Supporting Information is available in support of this manuscript and provides additional experimental data that complement the findings presented in the main text. The supplementary file includes (i) figures related to the optimization of the caffeine extraction process; (ii) graphs of adsorption isotherm models; (iii) FT-IR spectra of the [Nb­(Bez­(COO)_2_)]_*n*_ material after caffeine extraction (PDF)
