# Supplementary material for: A Niobium Coordination Polymer as an Efficient Sorbent for Caffeine Detection in Surface Water
Source: ACS Omega. 2025 Aug 21;10(34):38955–67. doi: 10.1021/acsomega.5c04894 (PMC12409570; doi:10.1021/acsomega.5c04894)
Supplement: Supplementary file 1 [file ao5c04894_si_001.pdf]

Supplementary Material

A Niobium Coordination Polymer as an Efficient Sorbent for Caffeine Detection in Surface Water

Emily Pachêco Squizatto[a,b], Iare S. Ribeiro[a], Marcos V. S. Pereira[a], Fábio Junior M. Novaes[b], Márcio J. da Silva[c], Luciano G. Moura[d], Gilberto R. da Silva Junior[d], Renê C. da Silva[d], Jemmyson R. de Jesus[a*]

[a] Research Laboratory in bionanomaterials, LPbio, Department of Chemistry, Federal University of Viçosa, 36570-900 Viçosa, Minas Gerais, Brazil.

[b] Analytical Chemistry Laboratory – LAQUA, Federal University of Viçosa, 36570-900 Viçosa, Minas Gerais, Brazil.

[c] Department of Chemistry, Federal University of Viçosa, 36570-900 Viçosa, Minas Gerais, Brazil.

[d] Department of Physics, Federal University of Viçosa, 36570-900, Viçosa, Minas Gerais, Brazil

*Corresponding author:

Prof. Jemmyson R. de Jesus

e-mail address: jemmyson.jesus@ufv.br

Table S.1 Coded matrix for multivariate analysis

Run	pH	Temperature (°C)	Adsorbent mass (mg)
1	-1	-1	-1
2	-1	-1	1
3	-1	1	-1
4	-1	1	1
5	1	-1	-1
6	1	-1	1
7	1	1	-1
8	1	1	1
9 (C)	0	0	0
10	-1.76383	0	0
11	1.76383	0	0
12	0	-1.76383	0
13	0	1.76383	0
14	0	0	-1.76383
15	0	0	1.76383
16 (C)	0	0	0

Table S.2 Results of caffeine extraction optimization using $[Nb(Bez(COO)_2)]_n$

Run	pH	Temperature (°C)	Adsorbent mass (mg)	Recovery (%)
1	-1	-1	-1	92
2	-1	-1	1	92
3	-1	1	-1	88
4	-1	1	1	45
5	1	-1	-1	88
6	1	-1	1	88
7	1	1	-1	59
8	1	1	1	67
9 (C)	0	0	0	80
10	-1.76383	0	0	45
11	1.76383	0	0	55
12	0	-1.76383	0	84
13	0	1.76383	0	76
14	0	0	-1.76383	79
15	0	0	1.76383	50
16 (C)	0	0	0	85

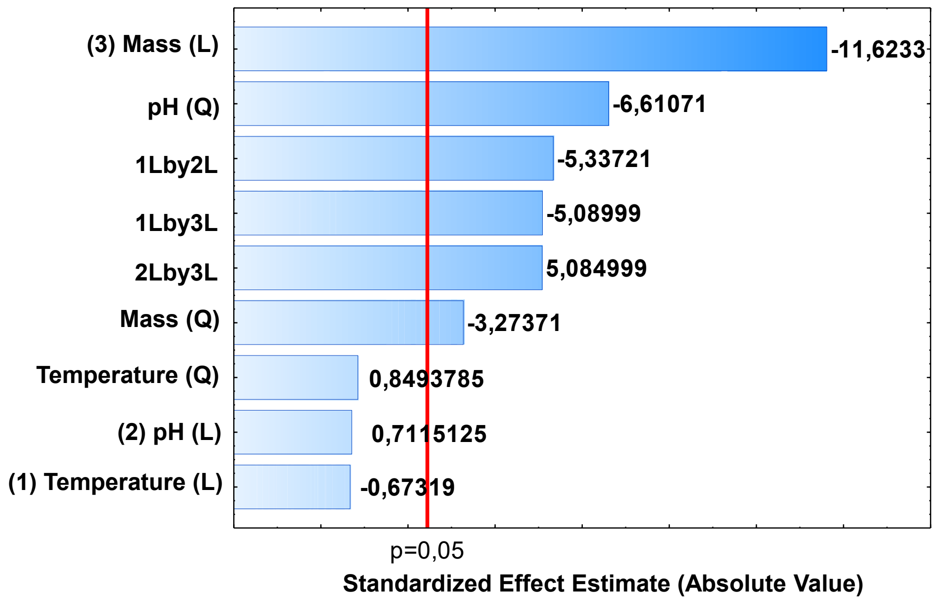

Figure S.1 Pareto chart highlighting the significant factors that influence the efficiency of caffeine extraction.

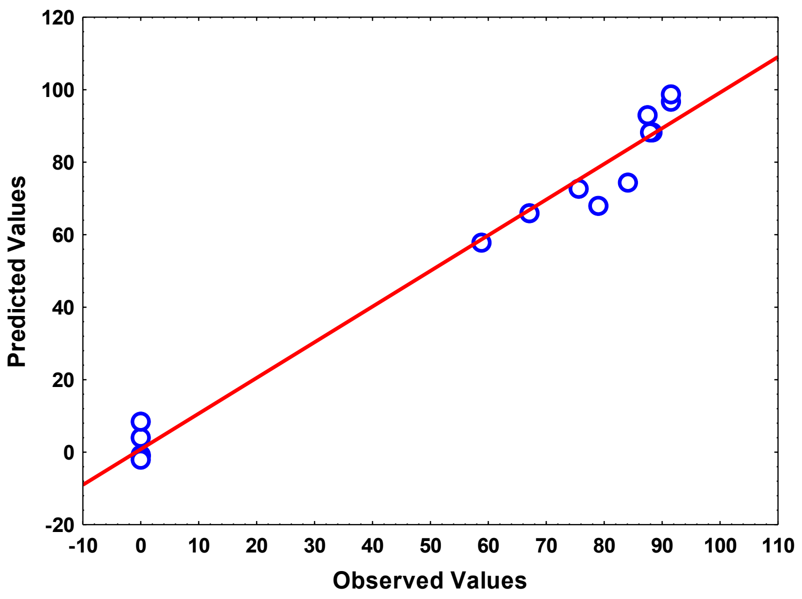

Figure S.2 Comparison between values predicted by the statistical model and observed values, demonstrating the good fit of the model and the efficiency of the caffeine extraction method.

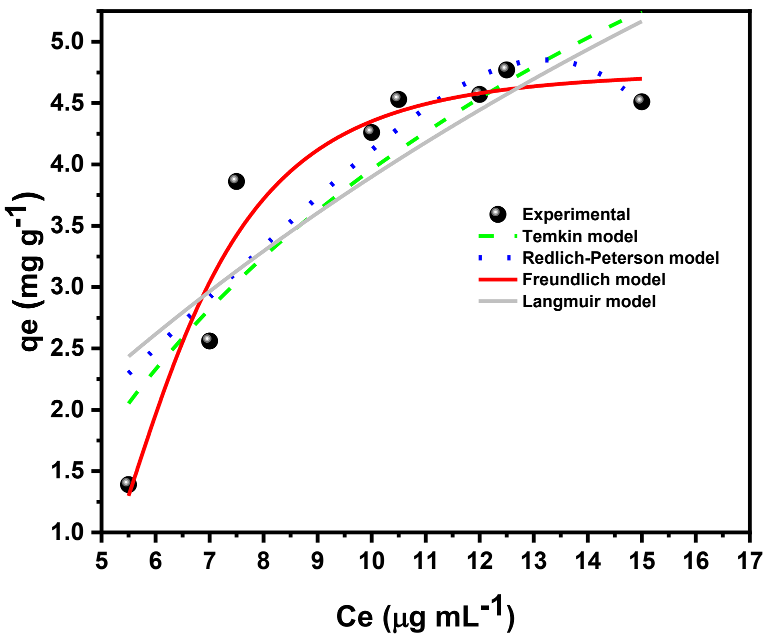

Figure S.3 Fitting of adsorption isothermal data used to investigate the adsorption mechanism and evaluate material performance.

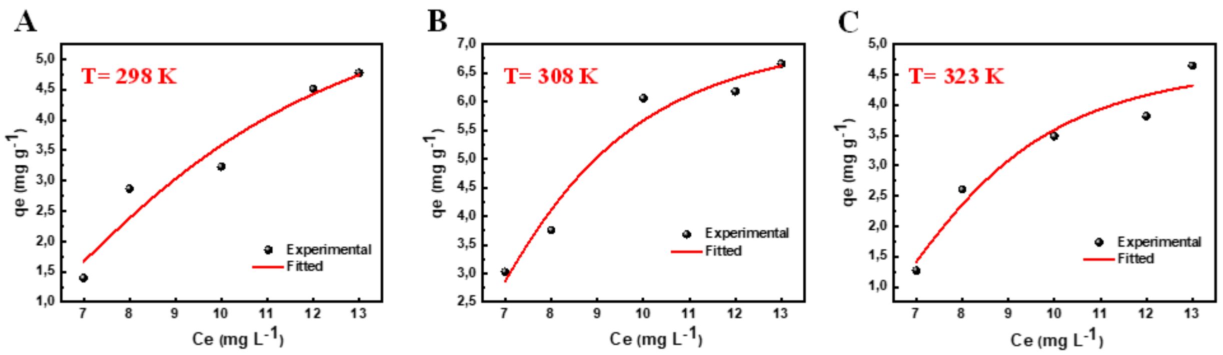

Figure S.4 Thermodynamic study of caffeine adsorption on $[Nb(Bez(COO)_2)]_n$ at temperatures of (A) 298, (B) 308 and (C) 323 K.

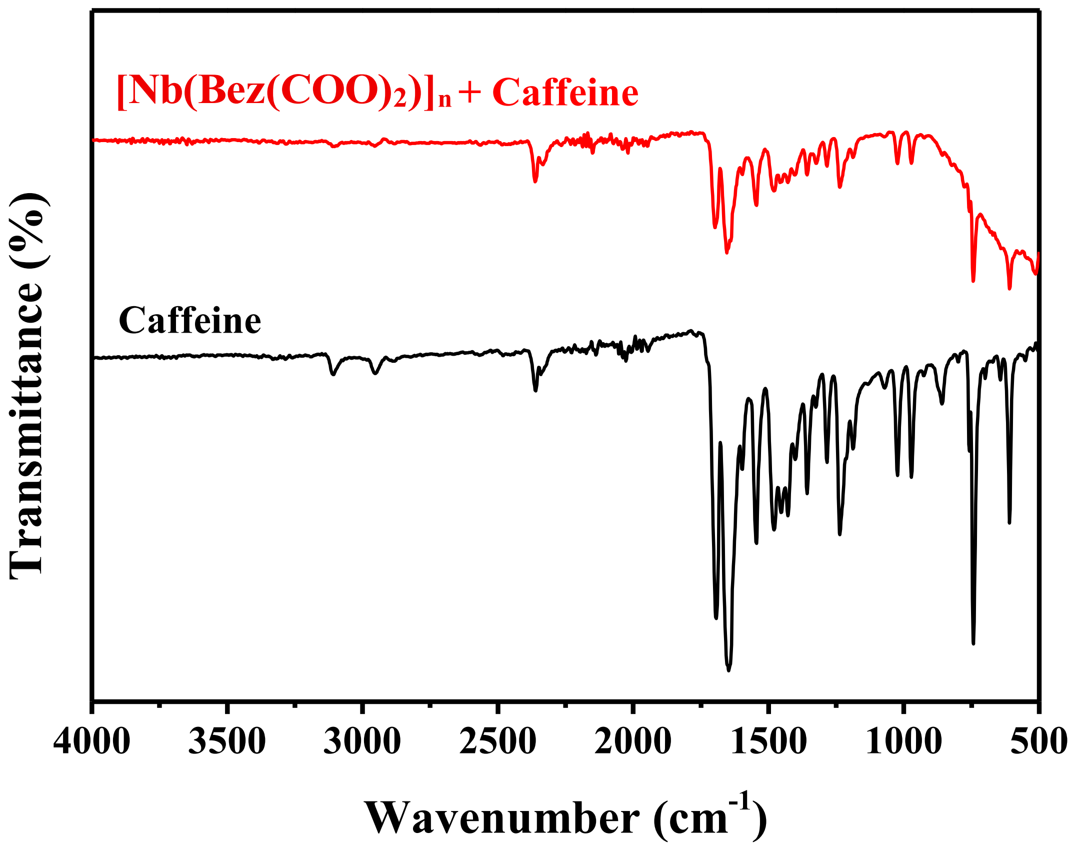

Figure S5 FT-IR spectra of pure caffeine and $[Nb(Bez(COO)_2)]_n$ after the extraction process confirming the adsorption of the analyte onto the material's surface**.**
